# Anomalous Supply of Anterior Compartment Muscles of the Arm by the Median Nerve in the Absence of the Musculocutaneous Nerve

**DOI:** 10.7759/cureus.81682

**Published:** 2025-04-03

**Authors:** KC Pradheep Kumar, Sanjukta Sahoo, Arthi Ganapathy, Prabhas R Tripathy

**Affiliations:** 1 Anatomy, All India Institute of Medical Sciences, Bhubaneswar, Bhubaneswar, IND

**Keywords:** brachial plexus, lateral cord, musculocutaneous nerve, nerve injury, variation

## Abstract

Anatomical diversity in the brachial plexus and its terminal branches in the right upper limb is described in this abstract. Variations in the brachial plexus are not unusual, but this case is notable since the musculocutaneous nerve is absent, and the median nerve has given branches to the structure in the anterior compartment as a result. In this study, we found that the musculocutaneous nerve does not innervate the anterior compartment of the arm; instead, the median nerve has taken on a broader role. In particular, the median nerve supplies the lateral aspect of the forearm, biceps brachii, coracobrachialis, and brachialis muscles. This instance emphasizes how important it is to comprehend and identify differences in neuronal architecture within the anterior compartment of the arm. Medical practitioners may find it helpful to know about these differences when diagnosing and treating individuals with upper limb disorders, as they have clinical implications.

## Introduction

The brachial plexus is the intricate network of nerves that innervates the upper limb. It comprises an organized hierarchy of roots, trunks, divisions, cords, and branches, each of which plays a distinct function in the movement of motor and sensory impulses to different arm muscles and locations. Differences in the brachial plexus are frequent and frequently highlight the extraordinary diversity of human anatomy [1].

Within the brachial plexus, an interesting and uncommon variant is the lack of the musculocutaneous nerve and the ensuing reorganization of the median nerve's branching in the anterior compartment of the arm [2]. The musculocutaneous nerve, primarily derived from the lateral cord of the brachial plexus (C5-C7), is responsible for innervating key muscles of the anterior arm compartment, including the brachialis, coracobrachialis, and biceps brachii. These muscles play essential roles in forearm flexion and supination, with the biceps brachii being a powerful supinator, especially when the elbow is flexed. The musculocutaneous nerve also provides sensory innervation to the lateral forearm via the lateral antebrachial cutaneous nerve. The musculocutaneous nerve in the arm is connected to the median nerve when the median nerve is small [3].

The lack of the musculocutaneous nerve and related changes in the median nerve are clarified by the case study of this uncommon anatomical variant. Our goal in presenting this uncommon but instructive finding is to give doctors and surgeons helpful knowledge that will help them better comprehend and manage the intricacies of the brachial plexus. This information is useful for surgical procedures involving the anterior compartment muscles of the upper arm and diagnosing and treating disorders affecting the upper limb. Understanding these anatomical variations is crucial for providing effective patient care and ensuring the success of surgical procedures.

## Case presentation

This case report describes a distinct anatomical variation discovered during a standard dissection of the anterior compartment of the right arm in a male cadaver of 55 years. The cadaver was legally procured through a body donation program/medical institution and embalmed using a standard formalin-based fixation method to preserve tissue integrity. Dissection was carried out following classical anatomical techniques, involving layer-by-layer dissection, meticulous separation of fascial planes, and careful tracing of neurovascular structures to document any deviations from typical anatomy. The differences found are unilateral and centred on the absence of the musculocutaneous nerve. In most cases, the nerve is essential for providing motor innervation to the muscles in the arm's anterior compartment. Nevertheless, the musculocutaneous nerve was conspicuously absent from this specific cadaver. This rare and intriguing finding revealed that, in the absence of the musculocutaneous nerve, motor innervation to its usual target muscles was provided by the median nerve instead.

**Figure 1:**
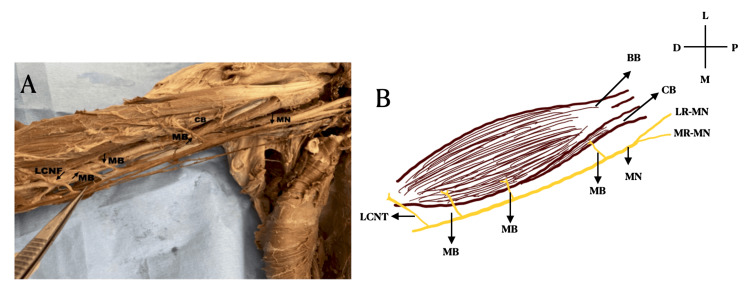
A) Cadaveric image in the absence of musculocutaneous nerve and muscular branches from the median nerve on the right side. B) Schematic image in the absence of musculocutaneous nerve and muscular branches from the median nerve on the right side MN: Median nerve, MB: Muscular branch, CB: Coracobrachialis, LCNF: Lateral cutaneous nerve of forearm, LR-MN: Lateral root of the median nerve, BB: Biceps brachi, MR-MN: Medial root of the median nerve, L: Lateral, M: Medial, D: Distal, P: Proximal

**Figure 2:**
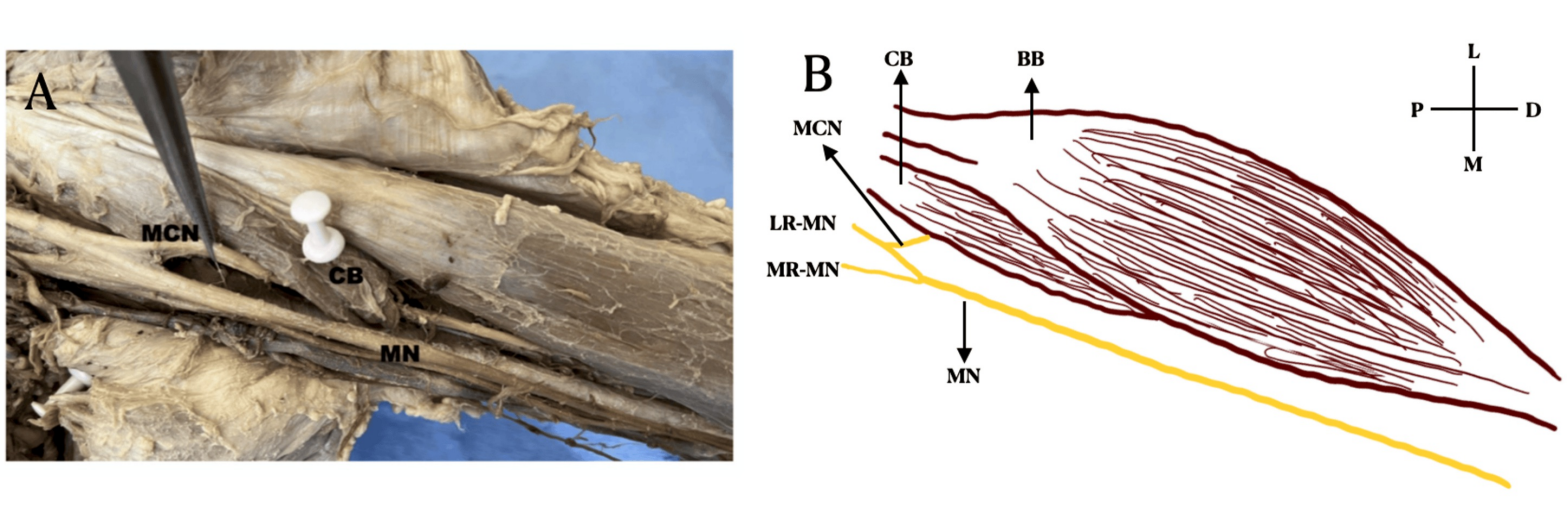
A) Cadaveric image of musculocutaneous nerve pierces coracobrachialis and continuing its normal course on the left side (white pinned structure showing coracobrachialis). B) Schematic image of musculocutaneous nerve pierces coracobrachialis and continuing its normal course on the left side MCN: Musculocutaneous nerve, MN: Median nerve, MB: Muscular branch, CB: Coracobrachialis, LCNF: Lateral cutaneous nerve of forearm, LR-MN: Lateral root of the median nerve, BB: Biceps brachi, MR-MN: Medial root of the median nerve, L: Lateral, M: Medial, D: Distal, P: Proximal

It was noted that the median nerve, which ordinarily innervates the muscles of the forearm and hand, split earlier in the upper arm than usual. These branches supplied the biceps and coracobrachialis muscles. At the point where it typically crosses the brachial artery, the median nerve splits into a second branch as it travels down the arm. This second branch was further split into two: the brachialis muscle was supplied by the first branch, and the second branch continued the lateral cutaneous nerve of the forearm. Notably, the median nerve's course and distribution beyond the cubital fossa stayed within normal parameters (Figure 1). An intriguing aspect of this case is the absence of any signs of accidental injury to the musculocutaneous nerve. The lack of this nerve could be attributed to the unmarked and intact skin of the arm, suggesting a congenital variation rather than trauma. On the left side, the median and musculocutaneous courses are normal (Figure 2).

## Discussion

This case report describes a unique anatomic variation in which the right arm's musculocutaneous nerve is completely absent. Because of this unusual observation, nerve fibers from C5 and C6 were rerouted to the median nerve via the lateral root. The median nerve was subsequently distributed to the muscles in the forearm's anterolateral cutaneous area and the anterior compartment of the upper arm. Such a departure from the usual anatomy is a rare occurrence, and it highlights the remarkable diversity of the human nervous system.

Although the median and musculocutaneous nerves have been shown to anastomose or connect, a complete absence of the musculocutaneous nerve is an uncommon finding.

Prasada Rao et al.'s study on 24 upper limbs showed communication between the musculocutaneous and median nerves in 33% of cases. This communication involved one or more connecting branches or a partial/complete fusion of the nerves. These findings suggest that variations in neural connections within the brachial plexus are relatively common [4].

In 26.4% of the 138 arms studied by Choi et al., connections were found between the musculocutaneous and median nerves. These relationships may result from branch fusion or the existence of one or more communicating branches. This study emphasises that these variations exist in a sizable percentage of cases [5].

Venieratos et al. identified 22 instances of communication between the median and musculocutaneous nerves in a study of 158 upper limbs. This finding supports the notion that such neural connections are more prevalent than previously thought [6].

Tsikass et al. identified a unilateral variation in a male cadaver, where the musculocutaneous nerve originated from the median nerve. This unique finding underscores the diversity of nerve branching patterns among individuals [7].

Le Minor [8], Nakatani et al. [9], and Ihunowo et al. [10] documented instances where the musculocutaneous nerve was completely absent. This represents the extreme end of the range of anatomical variations in the brachial plexus and is extremely uncommon. Unlike other cases that reported this anomaly on the left side or bilaterally, this case report describes a complete absence of the musculocutaneous nerve on the right side.

The embryological studies by Parchand et al. [11] and Hirasawa [12] explain the possible causes of the anatomical variation seen in the case report. It implies that the survival of a more rudimentary nerve pattern found in lower vertebrates may account for the loss of the musculocutaneous nerve and the subsequent adaptation of the median nerve to supply the lower arm muscles [11,12]. It has been observed that the median nerve in the thoracic limb usually has one trunk in lower vertebrates, such as amphibians, reptiles, and birds. Given that ontogeny recapitulates phylogeny, a developmental anomaly may cause the variation observed in this instance. As stated differently, this anatomical variation may be explained by the musculocutaneous nerve originating from the median nerve, which still exhibits a primitive embryological pattern.

Embryologically, the brachial plexus in the upper limb begins as a single radicular cone and eventually divides into ventral and dorsal segments. The ulnar and median nerves originate in the ventral segment, and the musculocutaneous nerve usually branches off the median nerve. This case provides strong evidence that the musculocutaneous nerve has a primitive embryological origin and shows how such primitive features can survive in a small subset of the population [13].

This case report highlights the rare absence of the musculocutaneous nerve and its replacement by the median nerve. It advances our knowledge and emphasizes how crucial it is to consider embryological and evolutionary factors when analyzing such anomalies in light of human anatomy. The case serves as a reminder of the human nervous system's intricate nature.

## Conclusions

The upper arm has a distinct pattern of nerve innervation due to the complete absence of the musculocutaneous nerve, resulting in the median nerve assuming its motor and sensory functions. Clinically, this variation is significant because an injury to the median nerve in the axilla would not only impact forearm and hand function but also compromise the innervation of key upper arm muscles, including the coracobrachialis, biceps brachii, and the medial portion of the brachialis. This could lead to elbow flexion weakness and a reduced ability to supinate the forearm, particularly when the elbow is flexed, as the biceps brachii play a crucial role in this movement. Supporting evidence from previous anatomical studies and embryological research further validates that such variations, although rare, reflect retained primitive nerve patterns. This underscores the clinical importance of recognizing these anomalies, particularly in surgical interventions, trauma assessments, and nerve repair procedures, where unexpected nerve pathways may alter the expected clinical presentation and treatment approach.
